# Impact of roadside burning on genetic diversity in a high‐biomass invasive grass

**DOI:** 10.1111/eva.13369

**Published:** 2022-03-27

**Authors:** Binyin Di, Jennifer Firn, Yvonne M. Buckley, Kate Lomas, Juli G. Pausas, Annabel L. Smith

**Affiliations:** ^1^ School of Agriculture and Food Sciences University of Queensland Gatton Queensland Australia; ^2^ 1969 School of Biology & Environmental Science Queensland University of Technology Brisbane Queensland Australia; ^3^ School of Natural Sciences, Zoology Trinity College Dublin The University of Dublin Dublin 2 Ireland; ^4^ Centro de Investigaciones sobre Desertificación (CIDE‐CSIC) Montcada Spain

**Keywords:** adaptation, asexual reproduction, fire ecology, fire management, invasive plants, landscape genomics, rapid adaptation, single‐nucleotide polymorphisms

## Abstract

The invasive grass–fire cycle is a widely documented feedback phenomenon in which invasive grasses increase vegetation flammability and fire frequency, resulting in further invasion and compounded effects on fire regimes. Few studies have examined the role of short‐term adaptation in driving the invasive grass–fire cycle, despite invasive species often thriving after introduction to new environments. We used a replicated (nine locations), paired sampling design (burn vs unburnt sites) to test the hypothesis that roadside burning increases genetic diversity and thus adaptive potential in the invasive, high‐biomass grass *Cenchrus ciliaris*. Between four and five samples per site (*n* = 93) were genotyped using the DArTseq platform, and we filtered the data to produce panels of 15,965 neutral and 5030 non‐neutral single nucleotide polymorphism (SNP) markers. Using fastSTRUCTURE, we detected three distinct genetic clusters with extremely high *F*_ST_ values among them (0.94–0.97) suggesting three different cultivars. We found high rates of asexual reproduction, possibly related to clonality or apomixis common in this species. At three locations, burnt and unburnt sites were genetically different, but genetic structure was not consistently related to fire management across the study region. Burning was associated with high genetic diversity and sexual reproduction in one genetic cluster, but with low genetic diversity and clonality in another. Individual SNPs were associated with longitude and genetic clustering, but not with recent fire management. Overall, we found limited evidence that roadside burning consistently increased genetic diversity and adaptive potential in *C. ciliaris*; evolutionary and breeding history more strongly shaped genetic structure. Roadside burning could therefore continue to be used for managing biomass in this species, with continued monitoring. Our study provides a framework for detecting fire‐related changes on a genetic level–a process that could be used as an early warning system to detect the invasive grass–fire cycle in future.

## 1 INTRODUCTION

In fire‐prone ecosystems globally, recurrent fire has shaped plant regeneration and demographic strategies, leading to fire‐dependent plant communities (Baker & Catterall, 2016; Keeley et al., 2011). Fire regimes—characterized by intensity, frequency, fire season and spatial characteristics of fire (Archibald et al., 2013; Gill, 1975)—can be altered by introduced non‐native plants when they change fuel properties (e.g. increased biomass) and when fire promotes their establishment and growth (Fusco et al., 2019; Gaertner et al., 2014). This can result in a positive ecological feedback, whereby the invasion of a non‐native plant increases fire frequency and/or intensity (Brooks et al., 2004; Rossiter et al., 2003), sometimes beyond the level where native vegetation can recover (Bowman et al., 2014). This phenomenon has been particularly documented in high‐biomass grasses and is often termed the invasive ‘grass–fire cycle’ (D’Antonio & Vitousek, 1992).

Most research on the invasive grass–fire cycle has focussed on site‐level changes in vegetation properties including, biomass, fuel connectivity, fuel moisture, plant architecture and trophic interactions (Gorgone‐Barbosa et al., 2015; Grigulis et al., 2005; McDonald & McPherson, 2013; Rossiter et al., 2003; Setterfield et al., 2013; St. Clair & Bishop, 2019). Invasive grasses have also been shown to pose a selection pressure on traits of the resident native community (Leger & Goergen, 2017). Few studies, however, have examined signatures of short‐term adaptation in invasive species themselves and whether this has a role in driving the invasive grass–fire cycle. This is partly because fire‐adaptive traits are usually examined in the context of long evolutionary timescales (Keeley et al., 2011; Pausas et al., 2012). Any process that increases genetic diversity however—including fire—could increase the likelihood of local adaptation, leading to population establishment and expansion.

Two phenomena give reason to consider the role of contemporary adaptation in contributing to the invasive grass–fire cycle. First, it is becoming apparent that evolution of fire‐adaptive traits within species can occur on ecological timescales (tens of generations or fewer, Carroll et al., 2007). For example, changes in anthropogenic fire regimes have been linked to increases in heat and smoke stimulated germination in *Calluna vulgaris* (Ericaceae) (Vandvik et al., 2014). Recent (500 years) introduction of fire by human activity has driven adaptation of seed shape and seed coat thickness in *Helenium aromaticum* (Asteraceae), allowing greater seed survival during fire (Gómez‐González et al., 2011). Phenotypic (Pausas et al., 2012) and genetic (Moreira et al., 2014) evidence shows that *Ulex parviflorus* (Fabaceae) is more flammable under a regime of frequent fire on a decadal timescale, giving support to the contested hypothesis (Bond & Midgley, 1995; Bowman et al., 2014) that flammability is adaptive. Thus, there is a need to re‐examine the timescales at which fire‐related traits could undergo adaptation.

The second reason why adaptative potential should be considered in understanding the invasive grass–fire cycle is the widely reported phenomenon of rapid adaptation in invasive species (Lee, 2002; Prentis et al., 2008; Whitney & Gabler, 2008). Invasive species can overcome climatic constraints on morphological traits by rapidly adapting to new environments (van Boheemen et al., 2019). Repeated introductions and long‐distance dispersal by humans can release invasive plant species from demographic constraints, such as those imposed by the colonization–competition trade‐off (Catford et al., 2018). Thus, plants in their non‐native range can overcome biotic and abiotic barriers because they are not always constrained by the same biological and climatic forces that operate in their native range (Smith et al., 2020). These demographic changes are often reflected in the species’ genetic structure (Lee, 2002; Rius & Darling, 2014), meaning that genetic data can shed light on the potential for rapid adaptation in invasive plants.

Traits related to reproduction are likely to be under stronger selection pressure than vegetative traits because of their direct contribution to survival and fecundity, particularly in short‐lived species (Lloret & Vilá, 2003; Villellas et al., 2021). Reproductive mode is a key trait related to fire regimes, and there are obligate and facultative forms of seeding and resprouting among postfire regeneration strategies (Pausas et al., 2004). Changes in fire regimes can alter the composition of reproductive modes in a landscape, which can, in turn, alter fire patterns (Batllori et al., 2019; Saura‐Mas et al., 2010; Simpson et al., 2021). Within species that have both seeding and resprouting populations, predominantly sexual populations often have greater genetic diversity than resprouting populations, reflecting shorter generation times and higher population turnover (Ojeda et al., 2016; Pausas & Keeley, 2014; Segarra‐Moragues & Ojeda, 2010; Simpson et al., 2021). In invasive plants, fire could therefore increase the proportion of sexually reproducing individuals, leading to increased population‐level genetic diversity and consequently greater adaptive potential. Thus, adaptation of fire‐related traits in invasive plants could augment the grass–fire cycle by increasing fire‐tolerant or fire‐promoting lineages.

Adaptation to changing fire regimes is a process that could take several generations and multiple fires to establish. As this occurs, a shift in reproductive strategy, from largely clonal to largely sexual, might be accompanied by increased genetic diversity and increased adaptive potential. Detecting such a change could serve as an early warning system that the grass–fire cycle was developing. To determine whether we could detect, at the molecular level, fire‐related changes in reproductive mode and genetic diversity, we studied an invasive, high‐biomass grass, which is commonly managed with roadside burning and has multiple reproductive modes. *Cenchrus ciliaris* is a perennial grass which became highly invasive in Australia after its establishment as a pasture grass in the late nineteenth century (Marshall et al., 2012). The species can resprout after fire (Fensham et al., 2013), or regenerate from seed either apomictically or sexually (Kumar et al., 2019). There are strong genetic differences underlying these reproductive modes (Yadav et al., 2019). Thus, if fire changes the population from predominantly clonal to predominantly sexual, this should be reflected in population genetic structure.

We used a replicated, paired sampling design to test the hypothesis that roadside burning increases genetic diversity and thus adaptive potential in *C. ciliaris*. Specifically, we asked: (1) does fire affect spatial genetic structure? (2) Does fire increase genetic diversity? And (3) Does fire influence the mode of reproduction (sexual or apomictic)? Our overarching aim was to provide knowledge to land management agencies about the likelihood that fire could enhance the ability for *C. ciliaris* to tolerate or benefit from fire. Prescribed burning is commonly used to manage *C. ciliaris* biomass (Northern Territory Government, 2014) so understanding whether fire acts an adaptive pressure seems critical to ensure this management practice continues to reduce biomass and does not promote genetic changes that enhance the fire cycle.

## 2 MATERIAL AND METHODS

### 2.1 Study region and target species

**FIGURE 1:**
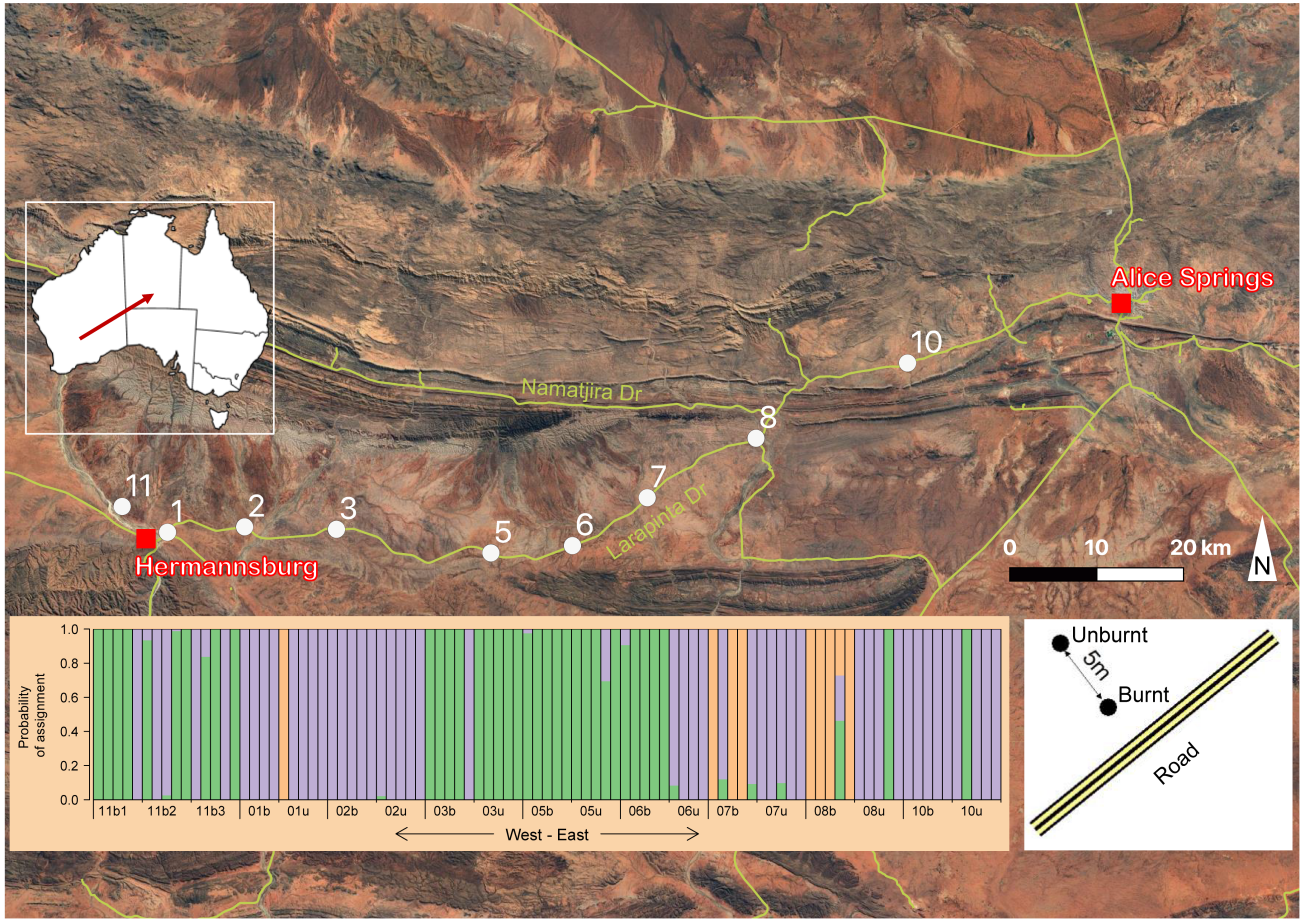
*Cenchrus ciliaris* DNA from 93 individuals was collected at nine sampling locations in central Australia. Each roadside location (i.e. all except location 11) consisted of a burnt site and a nearby unburnt site (lower right inset). At location 11, three sites were sampled where grasslands were managed by Indigenous rangers. The lower panel shows the probability of assignment of each individual plant to three genetic clusters identified by fastSTRUCTURE (*K* = 3), arranged by longitude

This study was conducted in hummock grasslands of central Australia, between Hermannsburg and Alice Springs, North Territory (Figure 1). Dominant native grasses are *Triodia* species. The region is arid with an annual average rainfall of approximately 238 mm, average maximum temperatures of 36 °C in December and average minimum temperatures of 4°C in July (Bureau of Meteorology, 2021). Traditional (pre‐European) fires in grasslands of the region were small (100–300 ha) and spatially patchy, driven by Indigenous burning for land management (Bliege Bird et al., 2012). Over the past century, this form of management has declined, and fires are now larger (1000–6000 ha) and more homogenous (Bliege Bird et al., 2012). Large fires in arid grasslands typically follow two or more years of above average rainfall, and the interfire interval can range from two years to over 20 years (Edwards et al., 2008; Wright & Clarke, 2007). A common contemporary practice along government‐controlled roads in the region is preventative burning, whereby prescribed fire is used to reduce biomass of invasive plants and prevent large wildfires (Northern Territory Government, 2014).

*Cenchrus ciliaris* L. (Poaceae) (buffel grass) is a perennial C_4_ grass, native to Africa, India and the Middle East, which has become invasive in the Americas and Australia (Marshall et al., 2012). In Australia, eleven official cultivars have been introduced and it has been widely used as a commercial pasture grass, for erosion control and for land rehabilitation (Marshall et al., 2012; Miller et al., 2010). The earliest account of arrival of *C. ciliaris* in Australia was ‘accidental’, reputedly in Afghan camel saddlery, between 1870 and 1880, including at Wallal on the north‐west coast of Western Australia (Marriott, 1955). The species is highly invasive in central Australia and has negative impacts on biodiversity (Read et al., 2020; Wright et al., 2021), including reducing the cover and diversity of native plant species (Franks, 2002; Jackson, 2005; Marshall et al., 2012) and altering faunal community composition and diversity (Bonney et al., 2017; Smyth et al., 2009). *Cenchrus ciliaris* can have a persistent soil seed bank (2–4 years; depending on soil type), which can tolerate fire and germinate rapidly after erratic rain events in arid climates (Tinoco‐Ojanguren et al., 2016).

Invasion of *C. ciliaris* is often accompanied by increased biomass which has been associated with hotter and more severe fires (Butler & Fairfax, 2003; McDonald & McPherson, 2013; Miller et al., 2010). In arid central Australia, fire accelerated the negative impacts of *C. ciliaris* on native species (Schlesinger et al., 2013). Another arid‐zone study found that *C. ciliaris* increased the severity of fire effects on native species, but there was limited power to detect whether fire promote growth and spread of the grass itself (i.e. the feedback aspect was not supported) (Miller et al., 2010). A study in savannah woodlands found that *C. ciliaris* invasion was driven by propagule pressure and grazing, but not fire (Fensham et al., 2013), confirming fire as an appropriate control method (Grechi et al., 2014). Thus, while the negative environmental impacts of *C. ciliaris* are clear, the extent to which fire enhances invasion is unresolved and probably depends on the environment.

### 2.2 Study design and DNA sampling

In 2018, we established eight sampling locations along Larapinta Drive (Figure 1) that had been recently burnt under the government’s preventative roadside burning programme. Evidence of recent fire at each location was obvious, with charcoal and scorched plants clearly visible on the surface. Sampling locations were separated by a mean of 13 km (range 9–20 km). Each location consisted of a burnt site immediately adjacent to the road (b) and a nearby unburnt site (u), at least five metres from the burnt site, which was obviously unimpacted by the fire. No data were available on fire history, so we do not know if the burnt sites had been repeatedly burnt in the past, although it is possible given the frequency of roadside burning in the region. Our sampling regime aimed to examine the effects of in situ roadside burning which is common in central Australia and elsewhere (Milberg & Lamont, 1995). This approach has the benefit of revealing empirical effects of current management, but cannot separate effects of confounded roadside conditions such as enhanced seed dispersal from vehicles and turbulent airflow, and increased water run‐off and nutrient availability (Milberg & Lamont, 1995; Milton et al., 2015).

At each site, DNA from 5 individual plants was collected, except at sites 1b and 6u where only four individuals were collected. In the following year, 2019, we sampled a ninth location, 6 km from site 1 (location 11, Figure 1), consisting of three sites in grasslands invaded by *C. ciliaris*. This area was managed by local Indigenous rangers and was burnt in June 2018, a year with low rainfall and slow vegetation regrowth. Prior to this, rangers had not observed fire in the time they had been managing the land. DNA from five individual *C. ciliaris* plants was collected at each of these three sites. We included location 11 in our descriptive analyses, but not in our formal statistical models given its low level of replication and different management regime. The total number of samples analysed for the study was 93.

### 2.3 Genotyping

Samples were genotyped at Diversity Arrays Technology P/L (Canberra, Australia) using double‐restriction enzyme (PstI and HpaII) complexity reduction and high‐throughput sequencing (DArTseq). This method was developed for crop plants and has been widely used on polyploid genomes to detect DNA polymorphism with high reproducibility (Akbari et al., 2006). It uses high‐fidelity restriction enzymes to target low‐copy fragments (~98% markers mapped to single location in the genome), which minimizes sequencing problems related to homology such as paralogous loci (Akbari et al., 2006) (we nonetheless tested for this, as described below). Total genomic DNA was extracted with a NucleoSpin 96 Plant II Core Kit (MACHEREY‐NAGEL) and purified using a Zymo kit (Zymo Research). DNA samples were processed in digestion/ligation reactions following Kilian et al. (2012) but substituting the single PstI adaptor for two adaptors corresponding to restriction enzyme‐specific overhangs. The PstI adaptor was modified to include Illumina sequencing primers and variable length barcodes following Elshire et al. (2011). Mixed fragments (PstI–HpaII) were amplified in 30 rounds of PCR using the following reaction conditions: 94°C for 1 min and then 30 cycles of 94°C for 20 s, 58°C for 30 s and 72°C for 45 s followed by 72°C for 7 min. After PCR, equimolar amounts of amplification products from each sample were bulked and applied to c‐Bot (Illumina) bridge PCR followed by single‐read sequencing on an Illumina Hiseq2500 for 77 cycles. Raw sequences were processed using DArTseq analytical pipelines (DArTdb) to split samples by barcode and remove poor‐quality sequences. Sequences of 69 bp were aligned to reference genomes of three Poaceae species (Rice_RGAP_v7, Sorghum_v8 and Switchgrass_v5) and bacteria (Bacterias_NCBI) with a BLAST E‐value of 5 ×10^−7^ and a minimum sequence identity of 90% using DArTseq proprietary software (DArTsoft). Replicate samples were processed to assess call rate (mean = 75%), reproducibility (mean = 99%) and polymorphic information content (mean = 29%).

### 2.4 SNP filtering and quality control

Starting with 69,799 SNPs that passed initial DArTseq quality control, we filtered the data using custom scripts developed by Smith et al. (2020) and modified for the current study (Figure S1). Filter 1 removed SNPs on the same sequence, monomorphic loci and SNPs with a low call rate (<50%). SNPs on the same 69‐bp sequence as another were removed to reduce the chance of disequilibrium from physical linkage, keeping the one with the highest call rate (Reynes et al., 2021). Filter 1 was applied before all further processing steps and analyses. Filter 2 removed SNPs with low minimum minor allele frequency (<5%), low reproducibility (<95%), linkage disequilibrium and putatively paralogous loci (*H*_obs_ > 80%, following Reynes et al. (2021) for partially clonal plants). We used the correlation between genotype frequencies (Chan, 2018) to test for linkage disequilibrium between each pair of loci and removed a locus if it was in a correlated pair (*r* > 0.75). The data comprised 20,995 SNPs after applying these filters (Figure S1).

### 2.5 Detecting loci under putative selection

Neutrality was an assumption underlying the population structure models we used. Thus, we investigated whether SNPs were putatively under selection using two individual‐level methods (PCAdapt and LFMM). LFMM is spatially and environmentally explicit and, in addition to testing model assumptions, we also used this test to investigate whether specific loci were related to the burn status of sampled individuals. If a variation at a locus was strongly related to whether individuals had experienced recent fire, it could elucidate the genetic basis underlying fire‐related genetic changes at a population level (Budde et al., 2017; Moreira et al., 2014). We conducted these tests after applying Filters 1 and 2 (20,995 SNPs, Figure S1). PCAdapt and LFMM both define background population structure as *K* principal components derived from individual genotypes (Duforet‐Frebourg et al., 2014; Frichot et al., 2013). In PCAdapt, each SNP is regressed against each principal component. LFMM uses the principal components as latent factors in a Gaussian mixed model, where the genotype matrix is modelled as a function of an environmental matrix (Frichot et al., 2013). Compared with tests designed for site‐level sampling designs, PCAdapt and LFMM are more reliable for species with complex, hierarchical population structure (e.g. multiple divergence events) and are less sensitive to admixed individuals and outliers in the data (de Villemereuil et al., 2014; Luu et al., 2017). We considered outliers identified in either of the two methods to be putatively under selection.

For PCAdapt and LFMM, we examined scree plots to determine *K* and assessed outliers against the first three and five components, respectively, that captured the majority of population structure in the data (Figure S2). We defined the LFMM environmental matrix using three environmental variables: fire category (a binomial variable indicating whether the site experienced recent fire), longitude (to account for the east‐west nature of our sampling design, Figure 1) and *K* (1–3, indicating three genetic clusters identified with fastSTRUCTURE, described below). To control for the false discovery rate, we calculated *q* values from *p* values and classed SNPs as outliers where *q* < 0.05. When combined with quality control filters our non‐neutral (putatively adaptive) data set comprised 5030 SNPs and, after filtering these loci, our neutral dataset comprised 15,965 SNPs (Figure S1).

### 2.6 Does fire affect spatial genetic structure?

All population structure analyses used our panel of 15,965 neutral SNPs (Figure S1), to comply with model assumptions about neutrality and linkage equilibrium. To assess genetic relationships among individuals, we first used fastSTRUCTURE (Raj et al., 2014). This model determines the number of genetic clusters in the data (*K*) that would maximize Hardy–Weinberg and linkage equilibrium. We investigated *K* = 1 to *K* = 11 and assigned each individual to a cluster based on the model complexity that maximized marginal likelihood and the model components used to explain structure in data (Raj et al., 2014). If fire affected spatial genetic structure, we expected to see genetic differences between burnt and unburnt sites within sampling locations, consistently across the study region. Thus, we visualized the results along a longitudinal gradient with samples grouped by burn category within location. *Cenchrus ciliaris* has strong genetic differences among reproductive modes, ploidy levels and accessions (Kharrat‐Souissi et al., 2011; Yadav et al., 2019), and we anticipated that fire effects might only be evident at certain levels of genetic hierarchy. Thus, we conducted hierarchical clustering using hclust in R (R Core Team, 2020) based on Euclidean distance between each pair of individual genotypes. To visualize the results in context of the *K* genetic clusters, we re‐projected the fastSTRUCTURE results along the hierarchical cluster dendrogram.

To further explore genetic differences among individuals, we conducted principal components analysis on neutral SNPs using the ade4 package (Dray & Dufour, 2007). We visualized *K* principal components that captured the majority of variation in the data. Finally, we conducted a site‐level analysis of *F*_ST_ in which burnt and unburnt sites within sampling locations were treated as separate ‘populations’. We calculated *F*_ST_ between all pairs of sites using diveRsity (Keenan et al., 2013) and conducted a mantel test in ecodist (Goslee & Urban, 2007) to assess whether *F*_ST_ was related to geographic distance. We conducted this test on the whole data set and then separately for sites within two of the three genetic clusters identified by fastSTRUCTURE (those with multiple sites containing four or more individuals: *K*1 = 9, *K*3 = 6). We also calculated *F*_ST_ values *between* the three genetic clusters identified by fastSTRUCTURE.

### 2.7 Does fire affect genetic diversity?

To investigate fire effects on genetic diversity, we examined site‐level and individual‐level genetic diversity and considered both neutral and putatively adaptive genetic diversity. Prior to analysis, we visualized our genetic diversity data (and data on reproductive mode, described below) with boxplots to summarize overall trends. Changes in neutral background genetic variation can reflect demographic processes such as admixture arising from nonrandom mating (Banks et al., 2013). On the other hand, if fire had cumulative effects on functional genes involved in reproduction and postfire regeneration, differences in adaptive genetic diversity might be apparent among sites.

We calculated site‐level allelic richness in hierfstat (Goudet & Jombart, 2015) separately for the panels of neutral and non‐neutral SNPs. Allelic richness was strongly correlated with expected heterozygosity (Pearson’s *r* > 0.97 for both data sets) but is standardized for sample size. We analysed the influence of fire category on allelic richness using linear regression in base R. Fire category was treated as a two‐level factor: roadside burnt and roadside unburnt (excluding location 11 with fewer replicates and a different management regime). To account for background genetic structure, we assigned each individual to one of *K* genetic clusters identified by fastSTRUCTURE and assigned site‐level *K* as the most common *K* among individuals at each site. We then fit an interactive model between fire category and *K* to test our hypothesis that fire would influence genetic diversity, while allowing this response to vary among genetic clusters. The model was fit separately to neutral and non‐neutral allelic richness data.

We calculated individual heterozygosity as the proportion of typed SNPs in each individual that was heterozygous, for neutral and non‐neutral markers separately. We analysed the influence of fire category (the two‐level factor, as above) on heterozygosity using a linear mixed‐effects model in lme4 (Bates et al., 2015), with site fitted as a random effect to account for spatial clustering of samples within sites. To account for background genetic structure, we assigned each individual to one of *K* genetic clusters identified by fastSTRUCTURE, using the *K* with the highest probability of assignment. The fixed effects part of the model was formulated in the same way as for site‐level allelic richness, with an interaction between fire category and *K*.

### 2.8 Does fire influence the mode of reproduction?

We calculated the kinship coefficient (the probability that two randomly sampled alleles from two individuals are identical by descent) with SNPrelate (Zheng et al., 2012), using the maximum‐likelihood estimator (Milligan, 2003). Kinship values of 0.5 and 0.25 indicate clones and full sibs, respectively. To investigate whether the probability of sampling asexually reproducing individuals varied across spatial scales, we assumed that kinship coefficients > 0.45 indicated clonal or apomictic individuals. We then calculated the proportion of pairwise kinship values in the data greater than this threshold in the whole data set, within locations (burnt/unburnt sites combined) and within sites. If fire increased rates of sexual reproduction, we expected to see a greater probability of sampling asexual individuals in the unburnt sites. Thus, we calculated the proportion of kinship coefficients > 0.45 at each site, as a rate of asexuality which we used to analyse the mode of reproduction.

To determine whether fire influenced the site‐level rate of asexuality, we used a beta regression generalized additive model with a logit link function in mgcv (Wood, 2011), suitable for continuous proportion data. To account for background genetic structure, we included site‐level *K* in the same way as for allelic richness and fit an interaction between fire category and *K*. This analysis was done using only neutral marker data as it relates to a demographic, rather than a selective, process.

In plants, *F*_IS_ in partially clonal lines has lower mean values, higher standard deviation and negatively skewed distributions compared with sexually reproducing lines (Reynes et al., 2021). Thus, to further investigate variation in clonality among the *K* genetic clusters identified by fastSTRUCTURE, we visualized the frequency distribution of *F*_IS_ (from hierfstat) and calculated the mean and interlocus standard deviation of *F*_IS_ for each cluster separately (Reynes et al., 2021). We did this using the partially filtered data (Filter 1:40,711 SNPs) to include loci with extreme *F*_IS_ that would be removed with following filters.

## 3 RESULTS

### 3.1 Loci under putative selection

**FIGURE 2:**
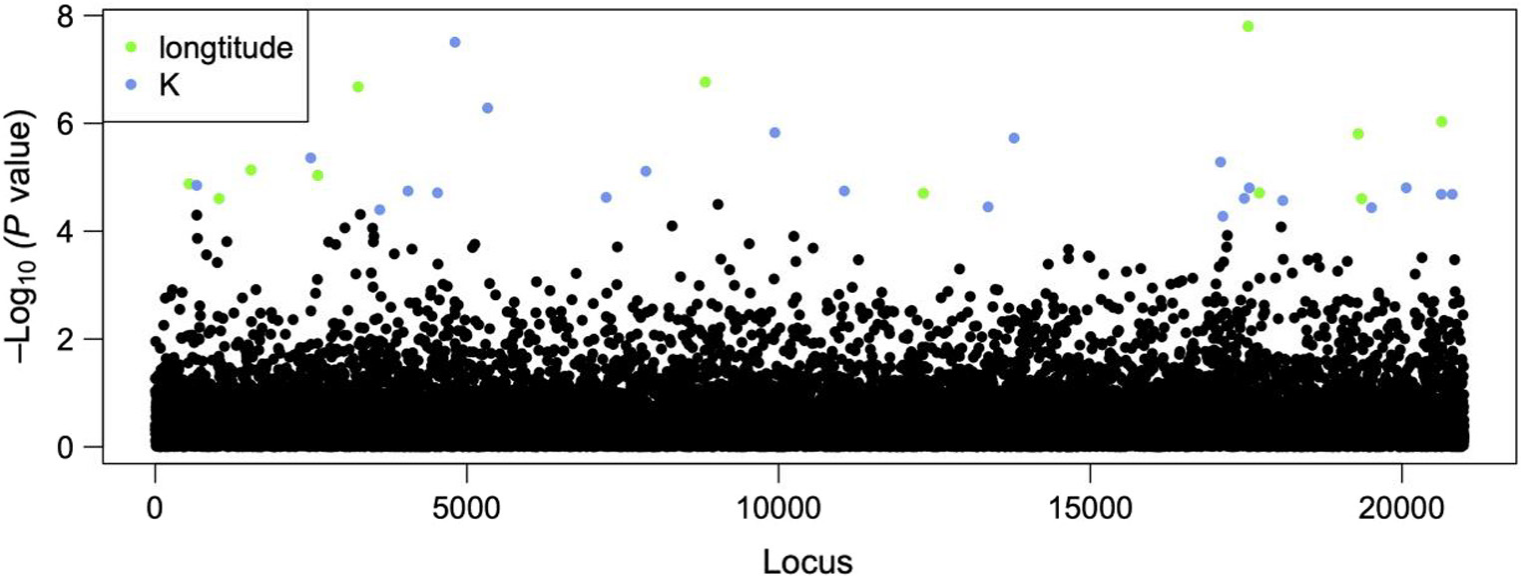
Results from LFMM outlier tests to determine loci putatively under selection. Three environmental variables were used in the analysis: fire category (a binomial variable indicating whether the site experienced recent fire), longitude and *K* (1–3, the three genetic clusters identified by fastSTRUCTURE). All 20,995 SNP loci used in the analysis are shown, with nonoutlying loci indicated in black. Outliers were related to longitude (12 loci, green points) and *K* (22 loci, blue points), but not to fire category

The two analyses used to detect loci under selection identified a total of 5030 outlier SNPs, 5005 with PCAdapt and 34 with LFMM. There was little overlap between methods (nine loci common to both methods, Figure S2), a phenomenon commonly reported in other studies (e.g. DeSilva & Dodd, 2020; de Villemereuil et al., 2014). In examining environmental associations with outlier loci, LFMM detected 12 SNPs related to longitude and 22 SNPs related to genetic structure defined by the three clusters identified by fastSTRUCTURE (Figure 2). There were no putatively adaptive loci related to fire category (i.e. whether individuals were from burnt or unburnt sites).

### 3.2 Does fire affect spatial genetic structure?

The number of genetic clusters among neutral *C. ciliaris* genotypes identified by fastSTRUCTURE (*K*) was between *K* = 3 (model complexity maximizing marginal likelihood) and *K* = 4 (model components used to explain structure in the data). We based our interpretation and downstream analyses on *K* = 3 (Figure 1) since *K* = 4 revealed only three individuals with genetic material from a fourth cluster (Figure S3). At the majority of roadside locations (1, 2, 3, 5 and 10), samples from burnt and unburnt sites were assigned to the same genetic cluster (Figure 1), indicating no detectable effect of fire on genetic structure at these locations. At three roadside locations (6, 7 and 8), individuals from the burnt and unburnt sites were assigned to two different genetic clusters, indicating differences in genetic structure between fire categories. At locations 7 and 8, the burnt sites consisted of a genetic cluster largely distinct from all remaining samples. Additional genetic structure at these sites was revealed in the *K* = 4 model (Figure S3). Outside of these burnt sites, only a single individual from site 1u was assigned to this cluster (cluster 1, indicated in orange).

**FIGURE 3:**
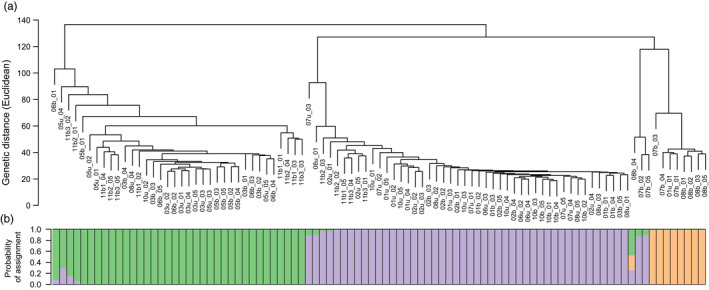
(a) Hierarchical clustering dendrogram showing genetic distance of among 93 *Cenchrus ciliaris* individuals; (b) Probability of assignment of each individual to three genetic clusters identified by fastSTRUCTURE (*K* = 3). This is the same data as in Figure 1 but arranged along the dendrogram to visualize the relationship between the two methods

Hierarchical clustering revealed higher‐level relationships corresponding largely to the three clusters identified by fastSTRUCTURE (Figure 3). The majority of individuals in the purple (*K*1) and orange (*K*2) clusters were genetically distinct, but more strongly related to each other than individuals from the green cluster (*K*3). Three individuals from sites 7b and 8b formed a distinct branch that was related at a higher level to the orange cluster (*K*2). All individuals except one in the orange cluster and this distinct branch were from burnt sites.

Both principal components analysis and *F*_ST_ revealed strong genetic structure in the data. Three principal components (PC) explained almost all of the variation in the data (89%) and showed strong differences among samples (Figure S4). Samples from some burnt sites separated along PC2, but these were the same individuals identified by fastSTRUCTURE (sites 7b and 8b) and there was no detectable relationship to fire history that was consistent among samples (Figure S4). Mean pairwise *F*_ST_ was 0.498 in the overall data set (range = −0.278 to 0.980), 0.022 in cluster *K*1 (range = −0.037 to 0.087) and 0.046 in cluster *K*3 (range = −0.004 to 0.212). There was no relationship between *F*_ST_ and geographic distance (isolation by distance) in either the overall sample (Mantel *r* = −0.03, *p* = 0.40, Figure S5a) or within clusters (*K*1 Mantel *r* = 0.04, *p* = 0.65; *K*3 Mantel *r* = 0.23, *p* = 0.75) (Figure S5b,c). *F*_ST_ between genetic clusters was very high: 0.97, 0.95 and 0.94 for clusters 1:2, 1:3 and 2:3, respectively, suggesting three different cultivars might have spread along the roadsides.

### 3.3 Does fire affect genetic diversity?

**FIGURE 4:**
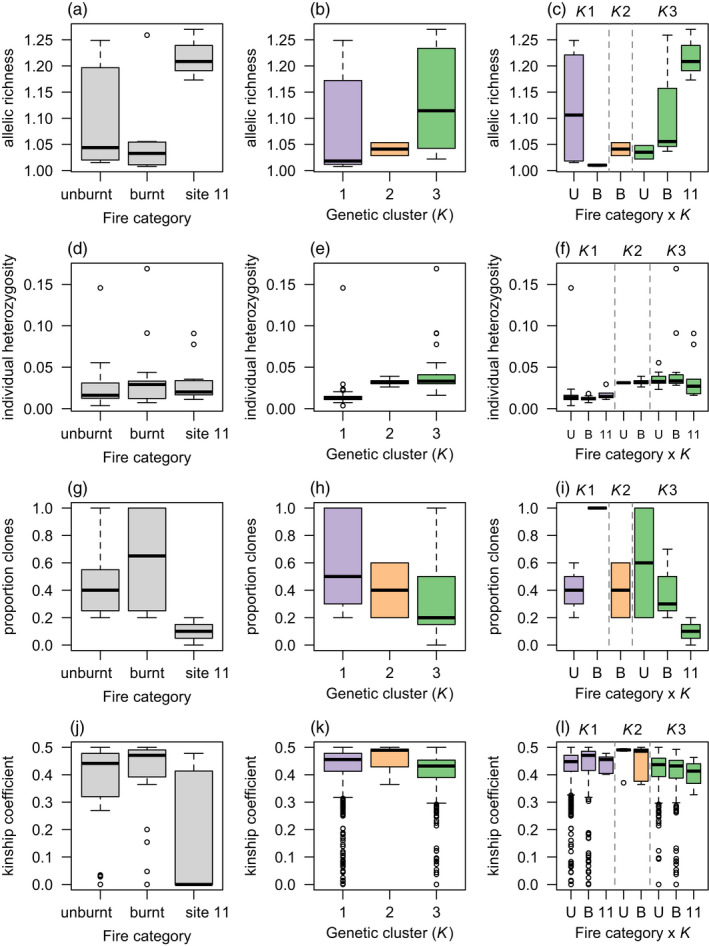
Summary statistics as boxplots (median, first and third quartiles and outliers) for response variables used to quantify genetic diversity and mode of reproduction in *Cenchrus ciliaris*, using neutral SNPs. (a–c) Site‐level allelic richness, (d–f) individual heterozygosity, (g–i) proportion of clones per site (kinship coefficient > 0.45) and (j–l) pairwise kinship coefficients. Each variable is shown for fire category: (roadside burnt, roadside unburnt and location 11) and *K* genetic clusters indicated by fastSTRUCTURE. Data are available online: https://zenodo.org/record/6342392

Summaries of the raw data showed that, compared with the roadside locations, location 11 had a higher site‐level allelic richness and lower rates of clonality, but similar levels of individual heterozygosity (Figure 4). There was substantial variation in genetic diversity among the *K* genetic clusters identified by fastSTRUCTURE and *K*2 had lower levels of intercluster variation in all response variables (Figure 4).

**FIGURE 5:**
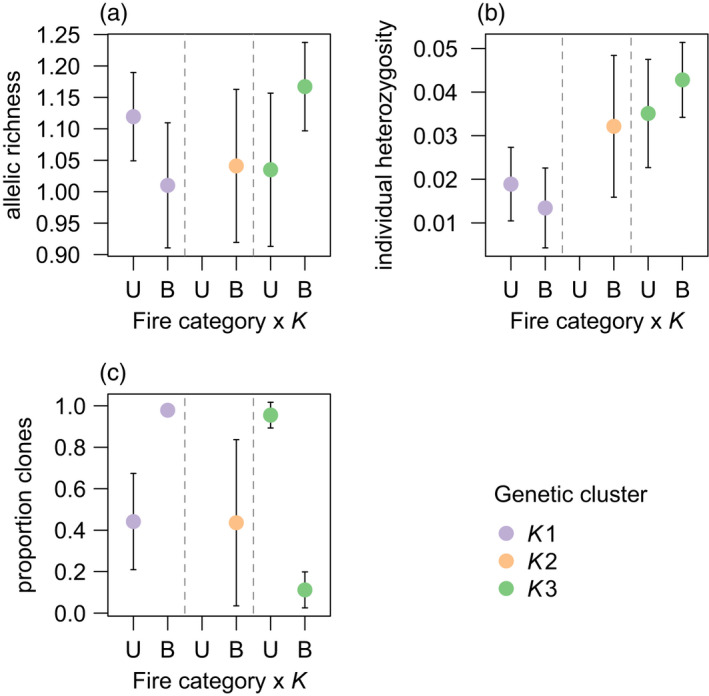
The interactive effect of fire category and background genetic structure (*K*) on neutral genetic diversity and the mode of reproduction in *Cenchrus ciliaris*. Model estimates and 95% confidence intervals are shown for (a) site‐level allelic richness, (b) individual heterozygosity and (c) the proportion of asexual individuals (clones) within sites (kinship coefficient > 0.45). A significant (*p* < 0.05) interaction was detected for allelic richness and proportion clones, while a significant main effect of *K* was detected for individual heterozygosity (Table 1). The colours correspond to the fastSTRUCTURE plots

There was a significant interaction between fire category and *K* for both neutral (*p* = 0.023) and non‐neutral (*p* = 0.024) allelic richness (Table 1). Compared with unburnt sites, neutral allelic richness at burnt sites was lower in *K*1 but higher in *K*3 (Figure 5a). Only burnt sites could be modelled for *K*2, which had intermediate levels of allelic richness (Figure 5a). The results for non‐neutral allelic richness were in the same direction and of a similar magnitude as for neutral allelic richness (Table 1). For both neutral and non‐neutral individual heterozygosity, the interaction between fire category and *K* was not significant, but there was a significant (*p* < 0.001) main effect of *K* (Table 1). Individual heterozygosity was lowest *K*1, highest in *K*3 with *K*2 showing similar levels to *K*3 (Figure 5b).

**TABLE 1:** Results from models examining the influence of fire category and background genetic structure on site‐level allelic richness, individual heterozygosity and the proportion of clones within each site

	Allelic Richness						Individual heterozygosity						Proportion clones		
	Neutral SNPs			Non‐neutral SNPs			Neutral SNPs			Non‐neutral SNPs			Neutral SNPs		
Model term	Coef.	SE	*p*	Coef.	SE	*p*	Coef.	SE	*p*	Coef.	SE	*p*	Coef.	SE	*p*
Intercept (Unburnt K1)	1.119	0.036		1.120	0.036		0.019	0.004		0.018	0.004		−0.235	−0.235	
Fire category (Burnt)	−0.109	0.062	0.940	−0.110	0.063	0.953	−0.005	0.006	0.902	−0.005	0.006	0.758	4.068	4.068	**<0.001**
*K* Genetic cluster			0.323			0.317			**<0.001**			**<0.001**			**<0.001**
*K*2	0.031	0.080		0.030	0.081		0.012	0.022		0.01	0.023		−4.091	−4.091	
*K*3	−0.084	0.072		−0.084	0.073		0.016	0.008		0.016	0.008		3.291	3.291	
Fire category x *K*			**0.023**			**0.024**			0.421			0.297			**<0.001**
Burnt *K*2	NA	NA		NA	NA		0.006	0.024		0.007	0.025		NA	NA	
Burnt *K*3	0.242	0.095		0.243	0.096		0.013	0.010		0.016	0.01		−9.195	−9.195	

Notes

Fire category included all roadside burnt and roadside unburnt sites and *K* represents the three genetic clusters identified by fastSTRUCTURE. Coefficients (Coef.) and standard errors (SE) are shown for each level within each term and *p* values are shown for each fixed effect in the model.

Bold values indicate significant effects (*p* < 0.05).

### 3.4 Does fire influence the mode of reproduction?

The proportion of pairwise kinship values in the data >0.45 (i.e. the rate of asexuality) was 0.20 in the whole data set, 0.32 within locations (burnt/unburnt sites combined) and 0.46 within sites (Figure S6). Thus, while the rate of asexuality was high at fine spatial scales (within locations and sites), asexual individuals were also sampled across sites that were separated by several kilometres. In analysing the rate of asexuality within sites, there was a significant interaction between fire category and *K* (*p* < 0.001) (Table 1). Compared with unburnt sites, the proportion of clones at burnt sites was higher in *K*1 and lower in *K*3, with intermediate levels shown for *K*2 (Figure 5c). All clusters had negatively skewed *F*_IS_ values and mean (and standard deviation) values less than zero: *K*1 = −0.631 (–0.746); *K*2 = –0.022 (–0.864); *K*3 = –0.123 (–0.824) (Figure S7). This indicates partial clonality in all clusters, a trend which was more pronounced in *K*1.

## 4 DISCUSSION

Our replicated study of the high‐biomass invasive grass *Cenchrus ciliaris* showed inconsistent effects of roadside burning on genetic structure, genetic diversity and the mode of reproduction. Some burnt sites were genetically distinct from paired unburnt sites, but this was not consistent across the whole study region. Burning was associated with high genetic diversity and sexual reproduction in one cluster, but with low genetic diversity and clonality in another. We found individual SNPs associated with longitude and genetic clustering, but not with recent fire management history. Thus, genetic structure appeared to be more to strongly related to evolutionary or breeding history than fire management. We were unable to quantify fire history at our study sites beyond very recent burning, and it is possible that too few fires had occurred to detect consistent fire‐related changes or that the long‐term fire history between burnt and unburnt sites were not strongly different. It might take repeated burning over several years for genetic changes to be detected.

Extremely high *F*_ST_ values (0.94–0. 97) among genetic clusters identified by fastSTRUCTURE and hierarchical clustering suggested three different cultivars of *C. ciliaris* in the data. High levels of *F*_ST_ can be driven by extremely low heterozygosity (Charlesworth, 1998) and this was a feature of our data (mean and range expected heterozygosity across sites = 0.127, 0.008–0.370). However, at least 10 different cultivars have been introduced into Australia on different properties or at different times (Marshall et al., 2012) and it is highly likely our data represent three different cultivars. We also found generally high rates of asexual reproduction, possibly related to clonal or apomictic reproduction, which is common in this species (Kumar et al., 2019; Yadav et al., 2019).

We found some influences of fire management on site‐level genetic diversity and reproductive mode in clusters *K*1 and *K*3, but the direction of these effects differed among clusters. Compared with unburnt sites, *K*1 had low allelic richness and high rates of clonality at burnt sites, while the opposite effect was shown for *K*3. In both these cases, increased allelic richness was associated with lower rates of clonality, a finding consistent with other studies of *C. ciliaris* in which sexually reproducing lines had higher genetic diversity (Kumar et al., 2019). Theory predicts that heterozygosity will increase with increasing rates of clonality (Balloux et al., 2003; Reynes et al., 2021) but, in our study, individual heterozygosity did not reflect patterns of clonality. Individual heterozygosity was generally highest in *K*3, which had variable levels of clonality (Figures 4 and 5).

There is a possibility that fire could have enhanced sexual reproduction in *K*3, leading to the observed higher genetic diversity at burnt sites. If variation in seed traits existed in the area, fire could reduce competition and favour individuals with traits that promote postfire reproduction, such as fire‐tolerant seed (Liyanage & Ooi, 2018; Moreira & Pausas, 2012). Seeds of *C. ciliaris* have been found to withstand temperatures of up to 100°C (Tinoco‐Ojanguren et al., 2016). Importantly, the variability observed in germination response to heat shock (Tinoco‐Ojanguren et al., 2016) means fire could act as a selection pressure on seeds better able to withstand fire, leading to the dominance of a more fire‐tolerant line. On the other hand, if there was variation in the capacity for postfire resprouting in *K*1, fire could enhance this process, leading to an overall greater level of clonality. This can occur when a species (or, by extension, a cultivar) has an evolutionary history that favoured drought tolerance with limited exposure to frequent or high‐intensity fire that would select for fire‐tolerant seed (Moore et al., 2019; Pausas & Keeley, 2014; Simpson et al., 2021). The contrasting results between the two genetic clusters make it difficult to draw any firm conclusions. Future work on this species will benefit from quantifying trait variation in relation to genetic diversity and fire regimes, across a wider range of cultivars and geographic areas (Bragg et al., 2015).

Our analysis of fire management using fastSTRUCTURE revealed that some recently burnt sites were genetically distinct from paired unburnt sites, but this pattern was not consistent among locations (Figure 1). At three locations, burnt and unburnt sites were genetically different and, at two of these locations (7 and 8), the burnt sites represented a genetic cluster distinct from the overall sample. This distinct genetic cluster (*K*2, indicated in orange) had intermediate levels of genetic diversity and clonality (Figures 4 and 5), so this genetic distinctiveness was not associated with genetic changes that would signal greater adaptive potential. In our study, fire was confounded with roadside location as burnt sites were, by design of the management programme, beside the road. This probably exposed them to more frequent propagule movement by seed sticking to vehicles or being moved through turbulent airflow at roadsides (Lemke et al., 2019; Rauschert et al., 2017), a hypothesis which was supported by the lack of isolation by distance in the *F*_ST_ data at a large spatial scale (100 km), even within genetic clusters. In a different bioregion of Australia, Fensham et al. (2013) found that propagule pressure, rather than fire, had a stronger effect on invasion in *C. ciliaris*, and we cannot separate this possibility from a potential fire effect at this stage.

The grassland that was managed by Traditional Owners and had experienced a recent fire (location 11) had higher site‐level genetic diversity than the roadside locations. Individuals from this area comprised two different genetic clusters, which probably led to the overall higher allelic richness. However, this site also appeared to have higher rates of outcrossing, with a lower rate of asexual reproduction and fewer clonal individuals. The different management regime at this site has not had profound effects on genetic structure as genetic clustering was not strongly distinct from the roadside locations.

While we did not find consistent effects of fire in our study, the finding of strong genetic structure within the overall sample highlights the importance of understanding cryptic genetic variation within invasive species (Ward et al., 2008; Wilson et al., 2009). Management decisions based on data from one cultivar might not be effective for another. If our study had been based only on data from *K*3, we might have concluded that fire would enhance sexual reproduction and adaptive potential. Our study shows, however, that we cannot draw such general conclusions. Our data also gives insights into the type of management appropriate for different populations. For example, managing a population with generally high rates of clonality (e.g. *K*1) using mechanical methods such as slashing or burning might not be as effective as for more sexually reproducing populations. Our study was limited to only one year of sampling per site and, even though our sampling spanned 100 km of central Australia, we sampled only three of at least 10 cultivars that have been introduced into the country. The species has an enormous distribution in Australia, covering all mainland states (Marshall et al., 2012). Thus, it would be beneficial to examine longer‐term effects of fire management on genetic diversity, in more cultivars across a broader geographic distribution.

We found limited evidence that roadside burning consistently contributes to increased genetic diversity and thus adaptive potential in *C. ciliaris*, with evolutionary and breeding history having a more dominant role in shaping population genetic structure. Roadside burning could continue to be used as a method for managing biomass in Central Australia and elsewhere but monitoring should continue as more generations and a longer exposure to burning might be required to see a change. Fire regimes are changing rapidly in Australia and globally (Abram et al., 2021), with severe negative consequences for biodiversity and ecosystem function (Kelly et al., 2020). The potential for invasive species to rapidly adapt to environmental conditions outside of their native range (Smith et al., 2020) means that repeated fire could assist invasion in future. Our study can serve as a framework for detecting such changes on a genetic level—a process that could be used as an early warning system to detect the grass–fire cycle in future.

## CONFLICT OF INTEREST

The authors declare no conflict of interest.

## Supporting information

Figure S1–S7

## Data Availability

Data and code supporting this article have been archived on Zenodo: https://zenodo.org/record/6342392.
