# Supplementary material for: Impact of roadside burning on genetic diversity in a high‐biomass invasive grass
Source: Evol Appl. 2022 Mar 27;15(5):790–803. doi: 10.1111/eva.13369 (PMC9108304; doi:10.1111/eva.13369)

SUPPORTING INFORMATION

The impact of roadside burning on genetic diversity in a high-biomass invasive grass

Authors

Binyin Di[1] 0000-0002-0289-8016

Jennifer Firn[2]

Yvonne M. Buckley[3] 0000-0001-7599-3201

Kate Lomas[2]

Juli G. Pausas[4] 0000-0003-3533-5786

Annabel L. Smith[1*] 0000-0002-1201-8713

[1] School of Agriculture and Food Sciences, University of Queensland, Gatton, 4343, Australia

[2] School of Biology & Environmental Science, Queensland University of Technology, Brisbane, 4000, Australia

[3] School of Natural Sciences, Zoology, Trinity College Dublin, The University of Dublin, Dublin 2, Ireland

[4] Centro de Investigaciones sobre Desertificación (CIDE-CSIC), 46113 Montcada, Valencia, Spain

* Correspondence: annabel.smith@uq.edu.au

Fig. S1. Flow diagram showing the steps used to filter SNPs for quality and neutrality. The number of SNPs removed at each step are indicated inside the square boxes. The diamonds indicate the number of SNPs retained after each filter.

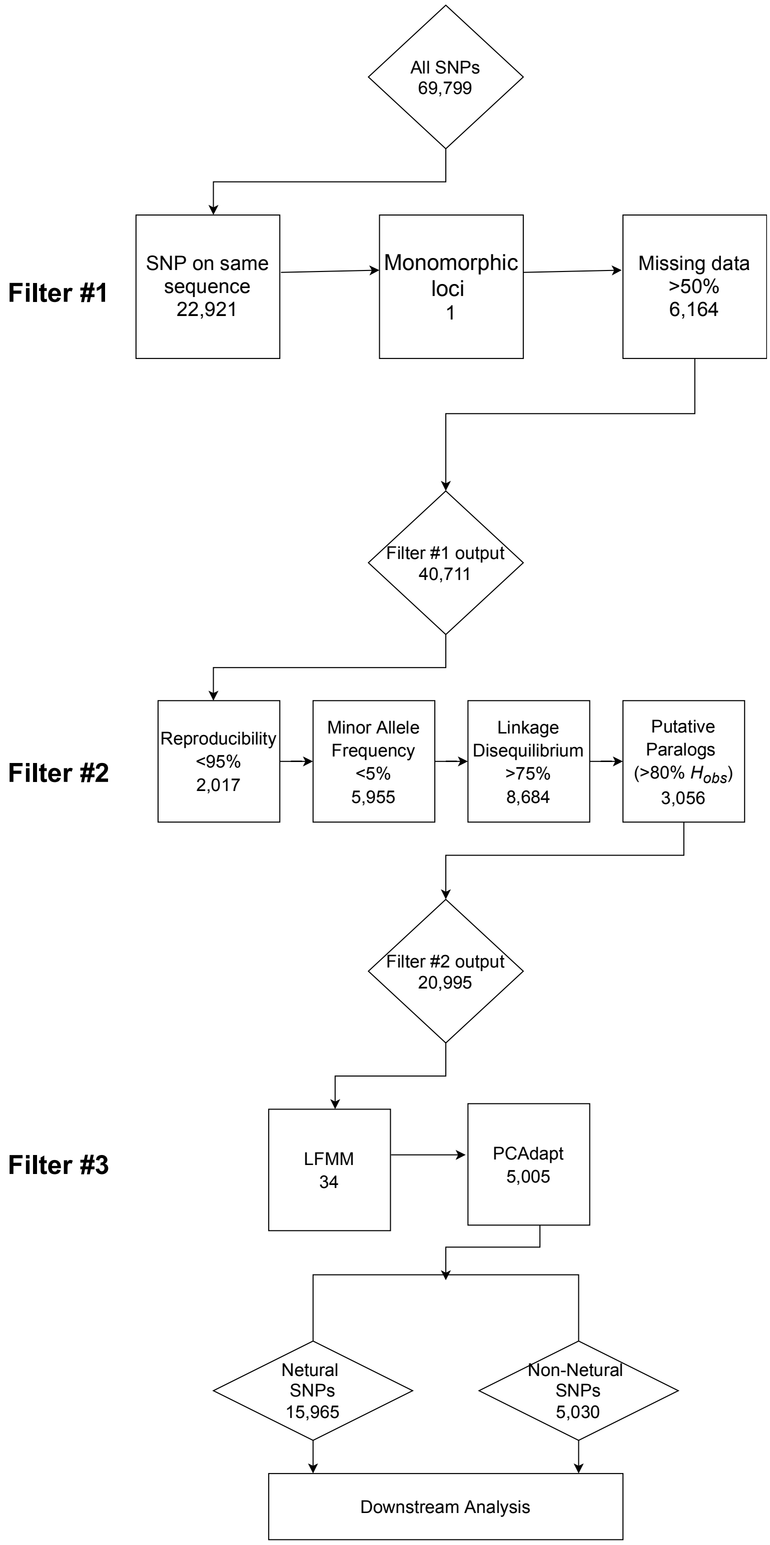

Fig. S2. Diagnostics and results from outlier tests to detect SNPs putatively under selection. (a) scree plot of principal components (PC) from PCAdapt (outliers were assessed against the first three PCs); (b) scree plot of PCs from LFMM (outliers were assessed against the first five PCs); (c) in total, 5030 SNPs were identified as outliers using both methods, with PCAdapt showing greater sensitivity to detecting outliers (5005) than LFMM (34). Nine loci were commonly identified as outliers using both methods. .

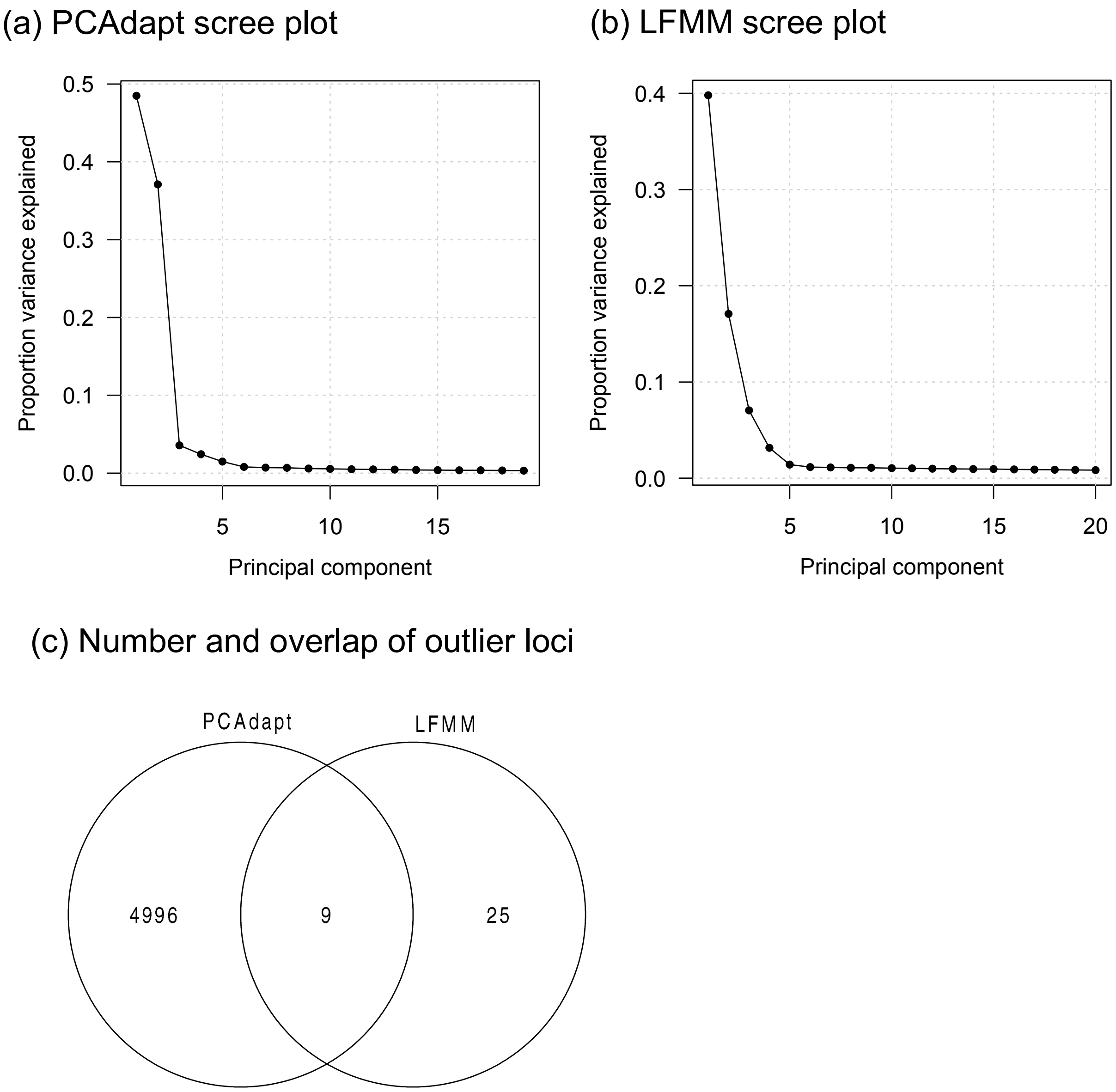

Fig. S3 Probability of assignment for 93 *Cenchrus ciliaris* individuals to genetic clusters identified by fastSTRUCTURE where $K = 4$, arranged by longitude. Model complexity which maximised marginal likelihood in the data was three (Fig. 1 and Fig. 3) and four model components were used to explain structure in the data ($K = 4$ shown here).

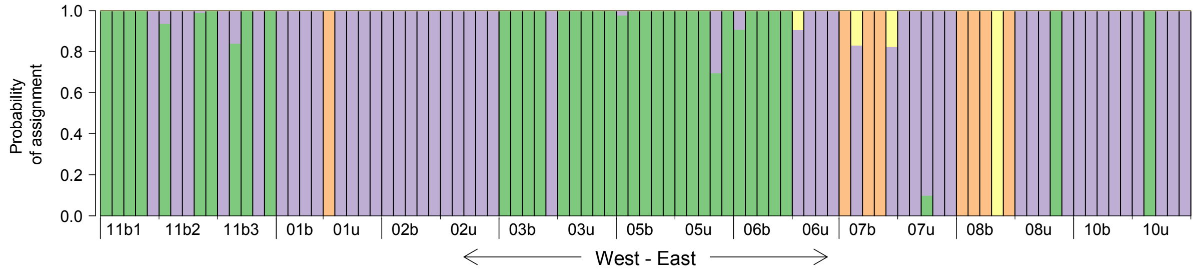

Fig. S4 Principal Components Analysis of neutral individual genotypes. (a) Scree plot showing the proportion variance explained for 22 principal components (PC). The first three PCs explained most of the variation in the data (cumulatively 89%). PC1 is plotted against (b) PC2 and (c) PC3 with individuals coloured by fire category: roadside burnt, roadside unburnt and location 11 (managed under a different regime).

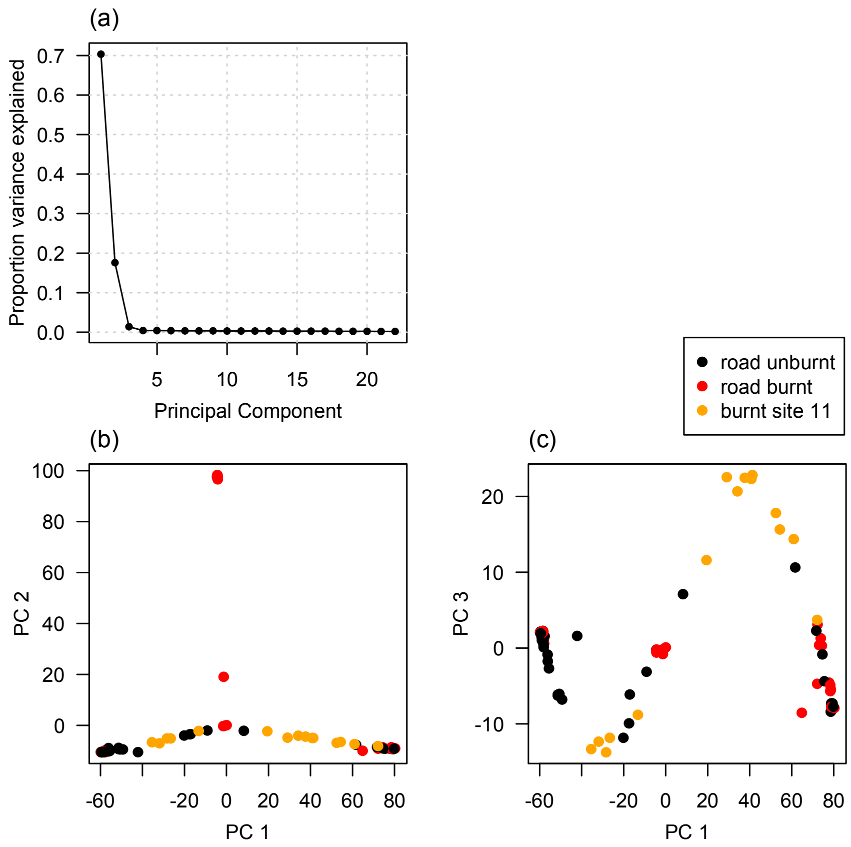

Fig. S5 The relationship between site-level F_{ST} and geographic distance. There was no indication of isolation by distance among (a) all sites (Mantel $r = -0.03$, $P = 0.40$), or within clusters ($K1$ Mantel $r = 0.04$, $P = 0.65$; $K3$ Mantel $r = 0.23$, $P = 0.75$). The colours in panels (a) and (b) correspond to the genetic clusters indicated on Fig. 1 and Fig. 3.

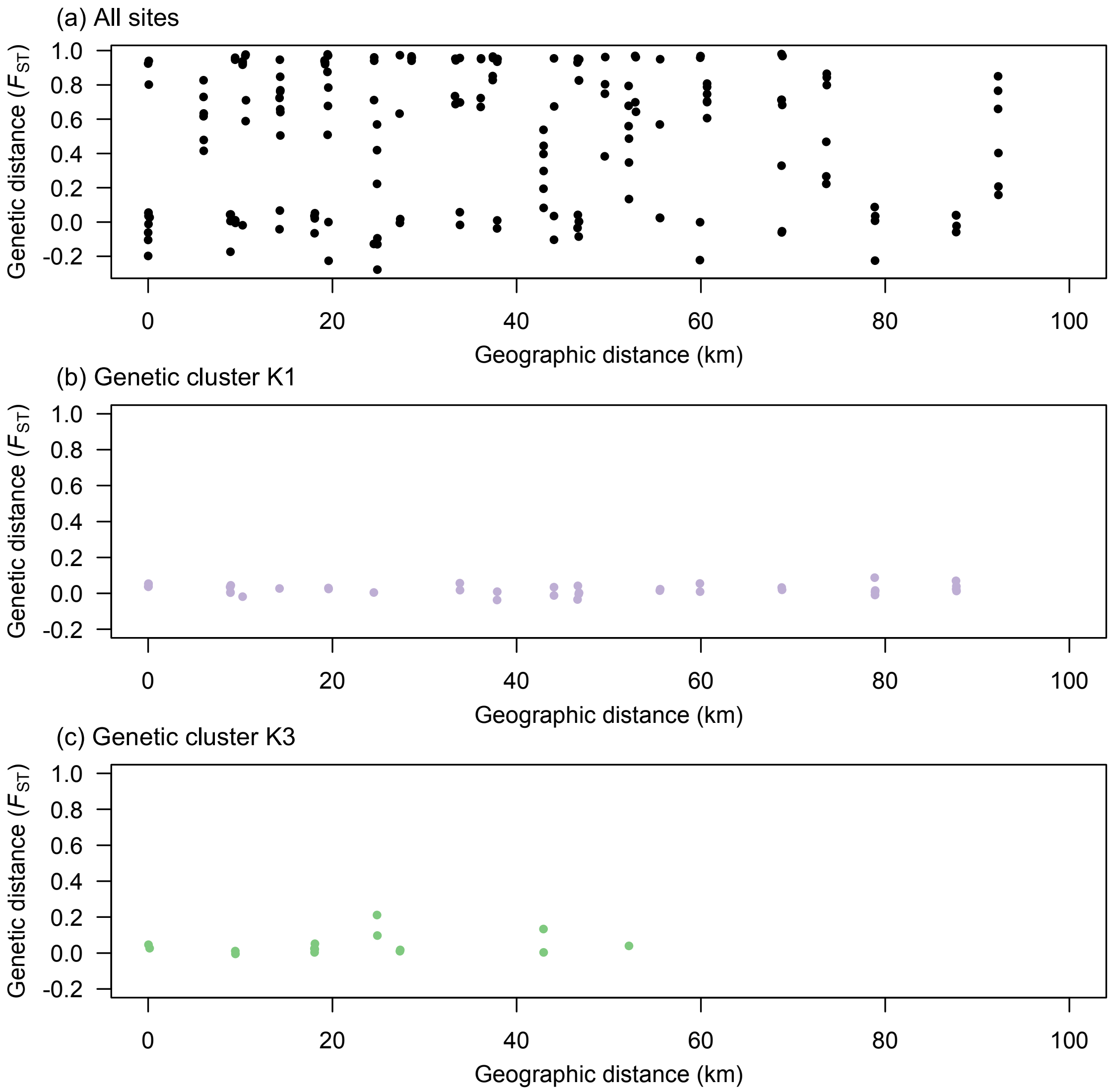

Fig. S6 The distribution of the kinship coefficient, showing the probability of sampling asexual individuals (kinship coefficient > 0.45) in (a) the whole data set, (b) within locations (burnt/unburnt sites combined) and (c) within sites.

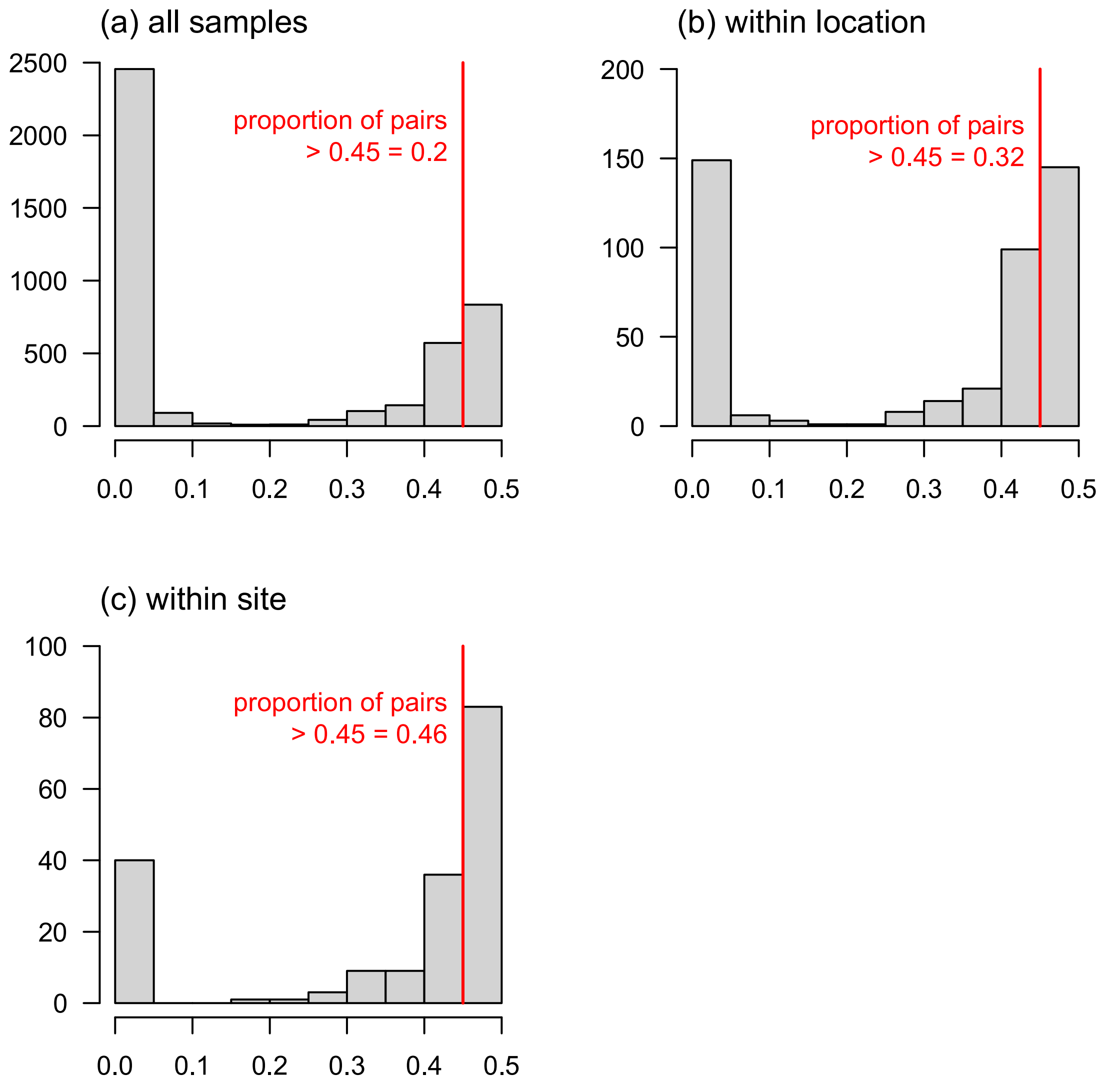

Fig. S7 The distribution of F_{IS} for each of three genetic clusters (*K*) identified by fastSTRUCTURE. All clusters had negatively skewed F_{IS} values and mean values less than zero (*K*1 = –0.631; *K*2 = –0.022; *K*3 = –0.123) indicating partial clonality in all clusters. This was more pronounced in *K*1, corresponding with our kinship analyses (see Fig. 4 in main document).

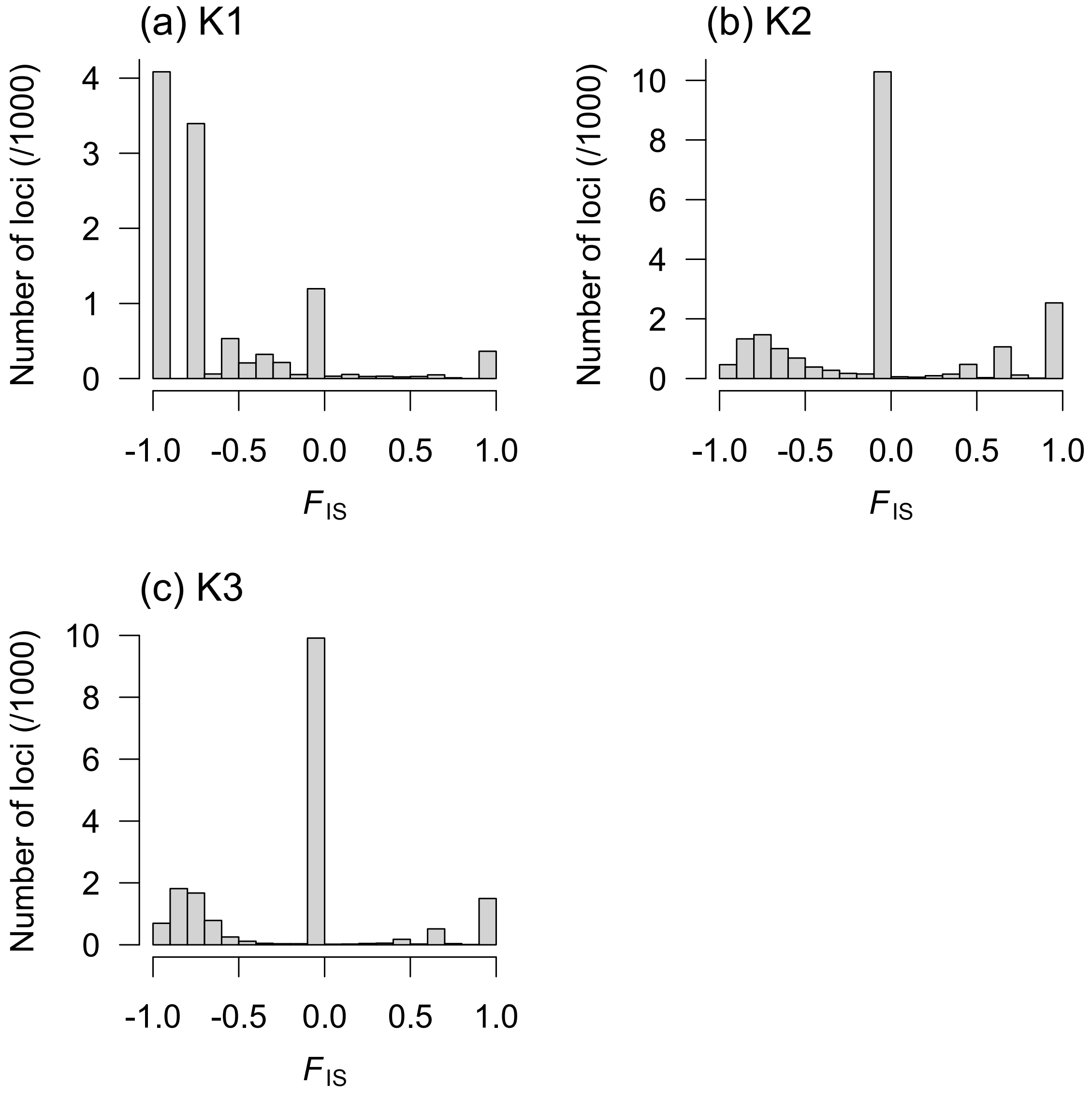

Supplement: Supplementary file 1 — Figure S1–S7 [file EVA-15-790-s001.pdf]
